# An overview of bacterial meningitis epidemics in Africa from 1928 to 2018 with a focus on epidemics “outside-the-belt”

**DOI:** 10.1186/s12879-021-06724-1

**Published:** 2021-09-30

**Authors:** Serge Mazamay, Jean-François Guégan, Neby Diallo, Didier Bompangue, Eric Bokabo, Jean-Jacques Muyembe, Nadège Taty, Tonton Paul Vita, Hélène Broutin

**Affiliations:** 1 Département de Microbiologie, Faculté de Médecine, Université de Kinshasa, Kinshasa, Democratic Republic of Congo; 2 MIVEGEC, Université de Montpellier, IRD, CNRS, 911 avenue Agropolis, BP 64501, 34394 Montpellier Cedex 5, France; 3 ASTRE, INRAE, Cirad, Université de Montpellier, Campus international de Baillarguet, 34398 Montpellier Cedex 5, France; 4 Chrono-Environnement, UMR CNRS 6249 Université de Franche-Comté, Besançon, France; 5 Département de Parasitologie-Mycologie, Faculté de Médecine, Université Cheikh Anta Diop (UCAD), Dakar, Sénégal; 6 Centre de Recherche en Ecologie et Evolution de la Santé (CREES), Montpellier, France

**Keywords:** Meningitis, Epidemics, Epidemiology, Pathogens, Time-series, Spatial analysis, In- and outside the meningitis belt

## Abstract

**Background:**

Bacterial meningitis occurs worldwide but Africa remains the most affected continent, especially in the "Meningitis belt" that extends from Senegal to Ethiopia. Three main bacteria are responsible for causing bacterial meningitis, i.e., *N. meningitidis* (Nm), *S. pneumoniae* and *H. influenzae* type b. Among Nm, serogroup A used to be responsible for up to 80 to 85% of meningococcal meningitis cases in Africa. Since 2000, other Nm serogroups including W, X and C have also been responsible for causing epidemics. This overview aims to describe the main patterns of meningitis disease cases and pathogens from 1928 to 2018 in Africa with a special focus on disease conditions “out-of-the-belt” area that is still usually unexplored. Based on basic spatio-temporal methods, and a 90-years database of reported suspected meningitis cases and death from the World Health Organization, we used both geographic information system and spatio-temporal statistics to identify the major localizations of meningitis epidemics over this period in Africa.

**Results:**

Bacterial meningitis extends today outside its historical limits of the meningitis belt. Since the introduction of MenAfrivac vaccine in 2010, there has been a dramatic decrease in NmA cases while other pathogen species and Nm variants including NmW, NmC and *Streptococcus pneumoniae* have become more prevalent reflecting a greater diversity of bacterial strains causing meningitis epidemics in Africa today.

**Conclusion:**

Bacterial meningitis remains a major public health problem in Africa today. Formerly concentrated in the region of the meningitis belt with Sub-Saharan and Sudanian environmental conditions, the disease extends now outside these historical limits to reach more forested regions in the central parts of the continent. With global environmental changes and massive vaccination targeting a unique serogroup, an epidemiological transition of bacterial meningitis is ongoing, requiring both a better consideration of the etiological nature of the responsible agents and of their proximal and distal determinants.

**Supplementary Information:**

The online version contains supplementary material available at 10.1186/s12879-021-06724-1.

## Introduction

Bacterial meningitis affects all countries of the world but primarily Africa especially the "Meningitis belt" that extends from Senegal near the Atlantic coast to Ethiopia and Somalia on the seashores of the Red Sea and the Indian Ocean [1]. Apart from epidemics, at least 1.2 million cases of bacterial meningitis are estimated to occur every year, 135,000 of which are fatal of these, ~ 500,000 and ~ 50,000 respectively are caused by meningococci with great heterogeneity in epidemiological patterns observed across the different affected countries [1]. Even when the disease is diagnosed early and adequate treatment is started, 8–15% of patients may die, often within 24 to 48 h after the onset of symptoms or may result in brain damage, hearing loss or disability in 10% to 20% of survivors [2].

Three main bacterial species are responsible for causing meningitis, *Streptococcus pneumoniae* (Sp), *Haemophilus influenzae* type b (Hib) and *Neisseria meningitidis* (Nm) most likely to cause major epidemics of cerebrospinal meningitis (CSM) [3]. Historically*,* Nm serogroup A (NmA) has been responsible for 80 to 85% of meningococcal meningitis cases observed in the meningitis belt [3–9].

Within the traditional meningitis belt, major epidemics occur every 5 to 12 years, with an attack rate of up to 1000 cases per 100,000 individuals [10]. Research studies carried out at the interface between epidemiology and climatology since the mid-XXth century have demonstrated the existence of a strong seasonal pattern of epidemics, which occurs during the dry season [10–17] i.e., from January to April. During this period, the dry winds of the Harmattan blow in Western Africa, carriers of dust and sand particles, may irritate the mucous membranes of the upper respiratory tract for people living in these regions. It should be noted that *Neisseria meningitidis* has the human oropharynx as an ecological niche. When the upper respiratory tract is colonized by the bacteria without causing disease, it is called a carriage state. On the other hand, when the oropharyngeal mucosa is attacked for example by the sand wind, the bacteria can cross into the general circulation and thus cause disease (a failing or dysfunctional relationship between the bacteria and the host). The bacterium is transmitted from person to person through droplets of respiratory or pharyngeal secretions and is often manifested by fever, stiff neck, headache, sometimes sensitivity to light and vomiting… [18–20].

The management of bacterial meningitis is both preventive (vaccines) and curative (antibiotics). Among the vaccines there are both polysaccharides and conjugated polysaccharide vaccines, including monovalent NmA, bivalent NmA and NmC, trivalent NmA, NmC and NmW, quadrivalent NmA, NmC, NmY and NmW, the conjugated vaccine Meningococcal NmC and the Nm serogroup B (NmB) vaccines which are protein-based and therefore potentially cover meningococci of other serogroups as well as not being comprehensive for serogroup B meningococci [2, 21, 22]. The recommended antibiotics are currently penicillin, ampicillin, chloramphenicol and ceftriaxone in monotherapy, especially in low-income countries [23–25].

The conjugate vaccine MenAfriVac was developed rapidly and has been deployed extensively since 2010 in all countries of the belt, with the goal of protecting populations against the most prevalent meningococcal meningitis serogroup, i.e., NmA [26–28]. Vaccination campaigns have been a great success particularly in Burkina Faso, Mali and Niger in 2010. Nearly 20 million people aged 1 to 29 years were vaccinated during these campaigns and the following epidemic season showed a dramatic reduction of NmA cases [29]. However, after the introduction of the MenAfrivac vaccine, other bacterial strains such as particularly NmW, NmC and *S. pneumoniae* have been responsible for developing new epidemic waves with a very high lethality rate between 5 to 6% [26]. In addition, some epidemics of meningitis have been shown to occur with irregular cycles within some countries of the belt [9, 30], suggesting that vaccine campaigns could have affected the spatio-temporal dynamics of meningitis spread or that ecological or evolutionary interferences could have happened between the different co-circulating bacterial strains causing meningitis.

At the same time, several other African countries located outside the so-called meningitis belt have faced sporadic but significant epidemics of meningitis [31–33]. A possible extension of the African meningitis belt to other African territories has raised the possibility that it may become necessary to extend vaccination programs beyond the previously prioritized targeted countries.

In one previous study, we show that an interplay of different geographical and environmental variables as latitude, longitude and socio-economic drivers are important to consider in the epidemiology of bacterial meningitis epidemics in DRC which is considered outside the meningitis belt [34].

In this study based on a 90-year time-series of suspected meningitis cases and death reported in the 53 different states in Africa by the World Health Organization (WHO), we provide a first descriptive analysis of continent-wide meningitis epidemics in order to characterize the existence of spatio-temporal patterns of transmission over this period. Data with pathogen identification is also illustrated. This work allows to extract several important conclusions about the circulation of meningitis pathogens and their recent African continent-wide spread, and to propose recommendations for the surveillance and control of these severe human infections all over Africa.

## Methods

### Study area

With its 54 independent countries and a population of approximately 1216 billion of people with a density of 40 inhabitants per km^2^, Africa is currently considered to be the second largest and most populated continent after Asia [35].

The topography of the continent is dominated by uplands cut in hard rocks and often lined with cliffs. The majority of the uplands can reach a height of 1500 m. There are also plains and higher mountains which are mainly concentrated in the eastern part of the continent.

The climate is determined by the crossing of the Equator line and the Tropics. There are four types of climate: (1) the *equatorial climate* (around the Equator) with two categories, a humid condition in the center and the west (1500 mm to over 2000 mm of water per year) and a drier one in the east (rainfall less than 1 mm); (2) the *tropical climate* (between the tropics, except the equatorial zone) with two subclimate types, a wet type (1200 to 1500 mm of rainfall per year) with 3 to 6 months of dry season and a dry type (between 500 to 1200 mm of rain per year) with 6 to 9 months of dry season; (3) the *desert climate* (north of the Tropics, i.e., the Saharan-Sudanian region) with less than 200 mm of rain a year; and (4) the *Mediterranean climate* at the top and bottom of the continent, with an average of 700 mm of water per year.

Each climate has a characteristic type of vegetation or biome. The equatorial climate is characterized by a dense rainforest, a developed fauna and flora. The vegetation of the tropical climate is distinguished from the others by the quasi-absence of forest or presence of the savannah or savannah-like ecosystems while that of the Mediterranean climate, by pine forests, scrubland and chaparral, and the desert climate by a dry to very dry, sparse or absent savannah [36, 37]. Meningitis has been associated with the sub-Saharan and Sudanian, desert-like and dry-savannah, environments, thus suggesting that bacterial meningitis is associated with the environmental conditions prevailing in those types of ecosystems [38, 39].

In the context of public health actions related to bacterial meningitis, the epidemiological surveillance currently carried out in sub-Saharan and Sudanian Africa in general and more particularly in the countries of the meningitis belt has the ultimate goal of contributing to reducing morbidity and mortality linked to these infections.

### Epidemiological data

We collected epidemiological data from two different sources:

#### Data from passive surveillance

We collected the annually numbers of suspected cases and deaths due to meningitis reported during the meningitis epidemics periods from the different national health systems to WHO using the different following official websites pages consulted on May 9, 2019:

https://apps.who.int/iris/handle/10665/229717?search-result=true&query=Meningitis+data+since+1928+in+Africa&scope=%2F&rpp=10&sort_by=score&order=desc. (Weekly Epidemiological Record 1930) (accessed 9.5.19).

https://apps.who.int/iris/handle/10665/72037 (accessed 9.5.19).

https://www.who.int/csr/don/archive/disease/meningococcal_disease/en/ (accessed 9.5.19). http://www.who.int/gho/epidemic_diseases/meningitis/epidemic_districts_text/en/ (accessed 9.5.19). http://www.safetravel.ch/safetravel2/servlet/ch.ofac.wv.wv203j.pages.Wv203ActualitesCtrl?action=afficheDetail&refActu=000437 (accessed 9.5.19).

https://www.who.int/emergencies/diseases/meningitis/epidemiological/en/ (accessed 9.5.19).

https://www.who.int/csr/resources/publications/meningitis/whoemcbac983.pdf?ua=1 (accessed 9.5.19).

https://apps.who.int/iris/bitstream/handle/10665/72037/bulletin_supp%20_Vol28.pdf?sequence=1&isAllowed=y (accessed 9.5.19).

We obtained data on suspected cases and deaths of meningitis between 1928 and 2018, as defined according to WHO guidelines [2] at the health area spatial scale in the different countries. We also collected additional data from previous research on meningitis epidemics in Africa from 1996 to 2006 (Kante, 2008). These data are freely available and accessible at: http://www.promedmail.chip/mailman/listinfo/promed and http://www.flutrackers.com/forum).

#### Data from laboratory analysis (confirmed cases)

We obtained the database of confirmed cases from 1928 to 2017 in Africa from WHO, which presents the information about all CSF samples received for meningitis diagnosis, including the date of sampling, number of positive samples, the pathogen identified by Latex test, culture or polymerase chain reaction (PCR). Due to the lack of data precision, only data from the years 2000 to 2017 were used in the present review. All data were collected on the WHO website at Iris/who/int and https://www.who.int/csr/don/archive/disease/meningococcal_disease/en/ (accessed 9.5.19).

### Shapefile data

Issued from WHO logiciel Healthmapper®.

### Data analysis

Spatial analyses of meningitis cases were performed with the softwares QGIS 2.14 (QGIS project, 2013) and Excel®.

- Graphics: Annual evolution of meningitis cases and deaths was performed with data from 1928 to 2018, using the Excel® software.
- Development of thematic maps: Maps showing cases distribution in Africa (meningitis belt or outside the belt) from 1928 to 2018 by states were performed using QGIS, using base maps extracted from Healthmapper® software produced by WHO. States with the highest attack rates ≥ 100,000 cases were considered to be the most affected by this infection.

We also developed maps showing the different spatial distributions for the different categories of pathogens causing bacterial meningitis according to positive meningitis samples collected in Africa from 2000 to 2017 (see above) by states, and performed using QGIS, using base maps extracted from Healthmapper® software produced by WHO. Annual time-series of meningitis and death cases were also studied using the software Stata ver. 14 ®.

## Results

**Fig. 1:**
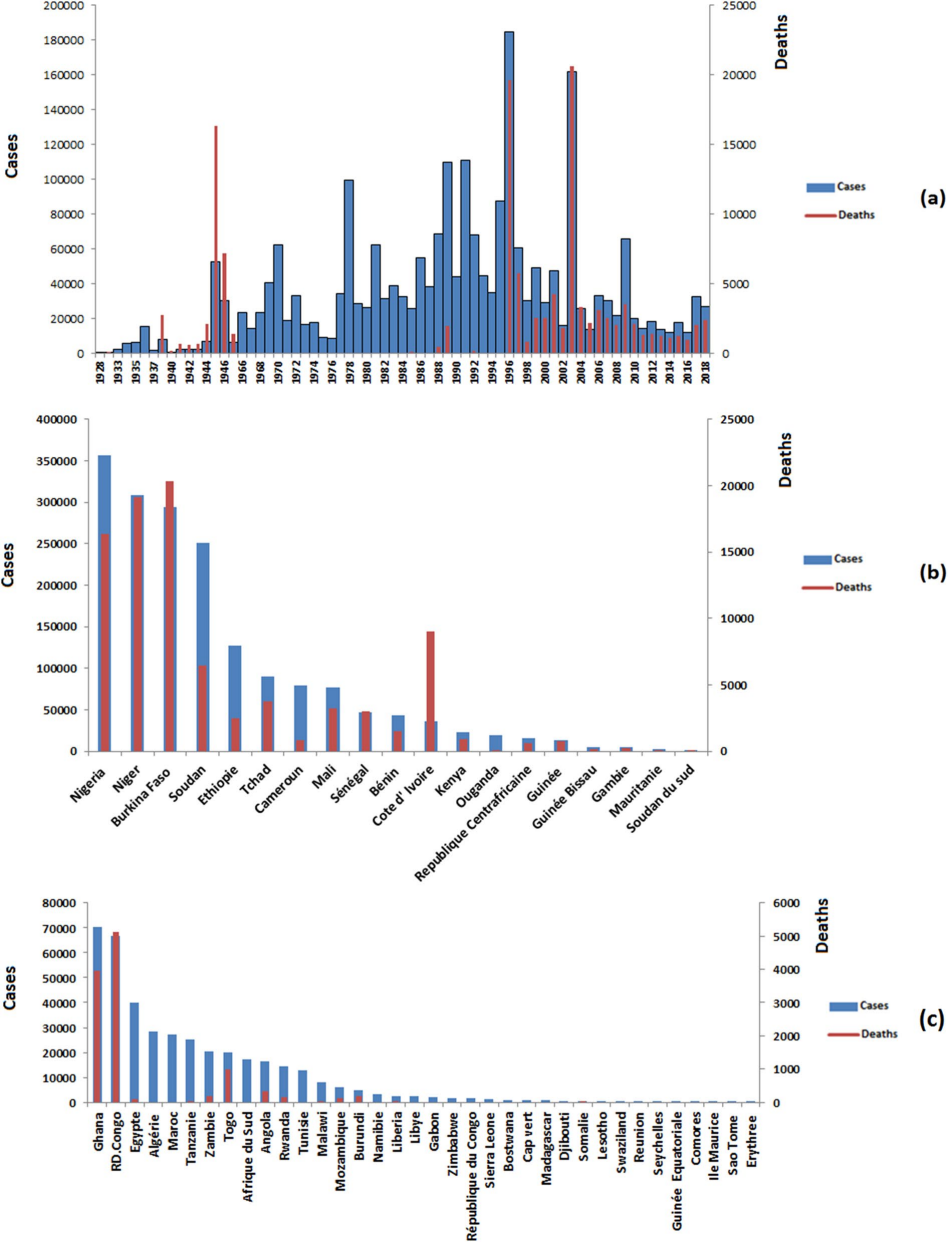
Annual meningitis cases (blue bars) and death (red line) in Africa, 1928–2018 (**a**), annual cases (blue bars) and deaths (red lines) of meningitis reported from 1928 to 2018 in countries within the belt (**b**) and outside the belt (**c**). Countries are ranked from left to right according to the number of cases reported

From 1928 to 2018, 2,628,283 cases including 151,808 deaths were reported to WHO-AFRO in the 53 African countries during the meningitis epidemics period, with a mean case-fatality rate of 5.77%. The highest case numbers were reported in 1996 (184,487cases) (see Fig. 1a). The mean incidence of meningitis cases in Africa from 1928 to 2018 was 216 per 100,000 inhabitants (SD ± 14.8).

Regarding the 20 countries located in the meningitis belt, Nigeria recorded the highest number of cases (552,821 cases, 21% of the total) during this study period followed by Sudan (359,434 cases, 13.6%), Burkina Faso (359,346 cases, 13.6%) and Niger (344,794 cases, 13.1%) (Fig. 1b). Out-of-the-belt, Ghana recorded the highest number of cases (70,508 cases, 2.6%) followed by the Democratic Republic of Congo (66,741 cases, 2.5%) and Egypt (40,014 cases, 1.5%) (Fig. 1c).

### Spatial dynamic trends (1928 to 2018)

**Fig. 2:**
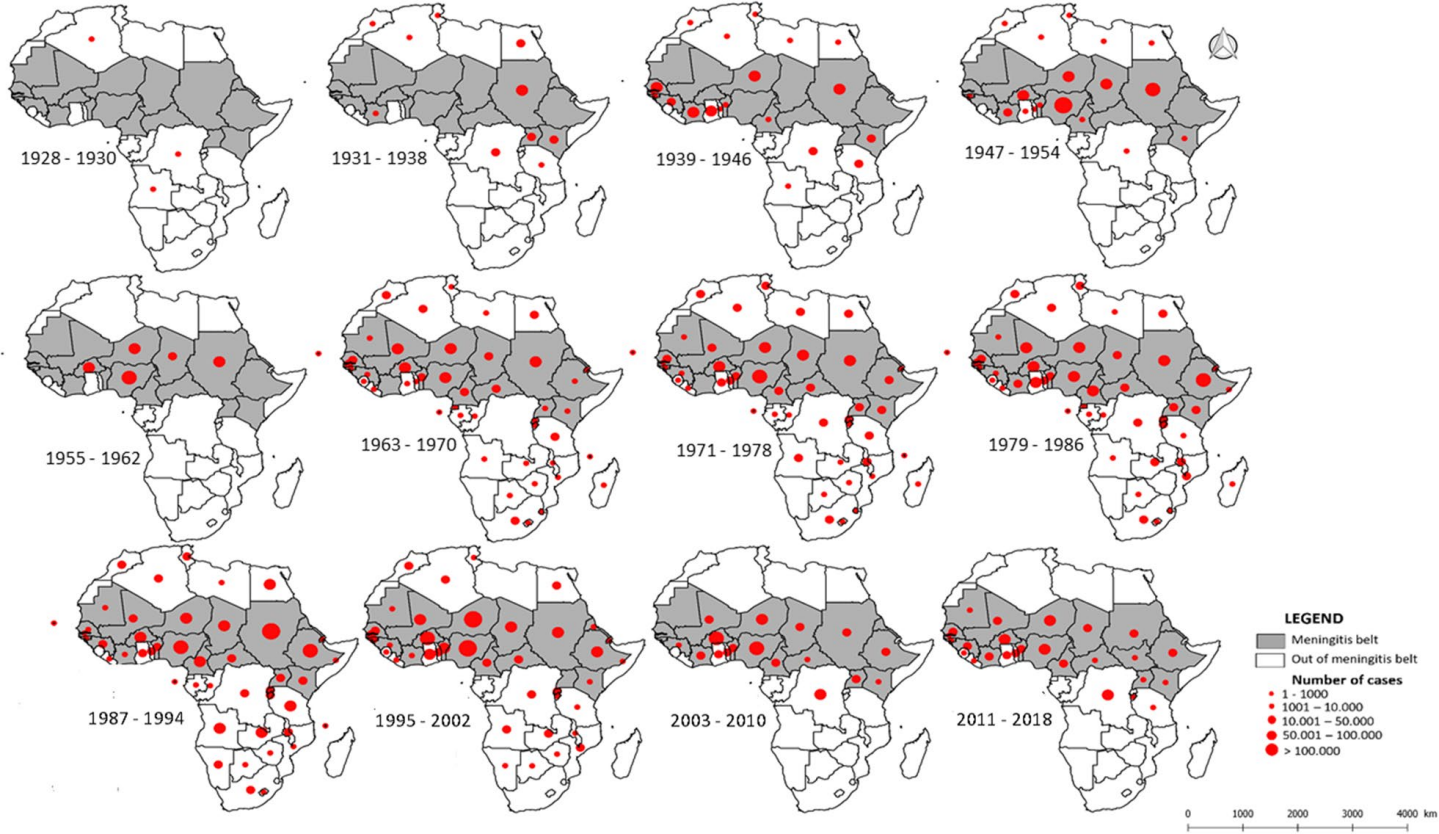
Spatial distribution of meningitis cases by country in Africa from 1928 to 2018 (Note that meningitis cases information is unavailable for the period 1948–1957). The areas in grey illustrate the different countries belonging to the meningitis belt

The spatial distribution of meningitis epidemics from 1928 to 2018, illustrated through 10-years periods of time (see Fig. 2), shows several important spatial trends. During the 1970s to the 1990s, meningitis epidemics were distributed widely on the African continent, with almost all the countries of the continent reporting cases. Towards the end of the 1990s and from the 2000s, meningitis epidemics appeared to decline in the south of the continent and in Northern Africa (see Fig. 2). From an epidemic point of view, the 1970s to the present show a near-universal African continent-wide distribution of major epidemics, with the late 1980s and 1990s harboring the greatest outbreaks of major meningitis epidemics. From the 2000s until now, epidemics seem less important and concentrated in the upper part of the meningitis belt and the central part of the continent (Fig. 2). From 2008 to now, Tanzania, DRC and Burundi located outside-the-belt are currently the countries reporting an important burden of meningitis cases (42,857/254,327 cases, or 16. 9%).

### Laboratory confirmation

#### CSF isolates

The distribution of 11,585 positive CSF samples is shown in Table 1.

**Table 1:** Confirmed cases of meningitis, Africa, 2000–2017

Pathogens	Number of positive CSF	Percentage
*Streptococcus pneumoniae*	2826	24.39
*Haemophilus influenzae*	362	3.12
*Neisseria meningitidis*	7747	66.87
*Neisseria meningitidis A*	1161	10.02
*Neisseria meningitidis B*	1	0.01
*Neisseria meningitidis C*	2771	23.92
*Neisseria meningitidis W*	2796	24.13
*Neisseria meningitidis X*	552	4.76
*Neisseria meningitidis Y*	6	0.05
Other *Neisseria meningitidis*	460	3.97
Other pathogens^a^	620	5.35
Positive Latex test^a^	30	0.26
Total of positive CSF	11,585	100.00

^a^Other pathogens include *Streptococcus* spp., *Streptococcus* group D, *Salmonella* sp., *Enterobacter* spp., *Citrobacter* spp.*, Staphylococcus aureus*, *Escherichia coli, Klebsiella* sp.*, Candida* sp*., Acinetobacter* sp.*, Listeria* sp*., Cryptococcus neoformans* and *Pseudomonas* sp. (Source: WHO)

Nm accounted for 66.87% of the total of meningitis cases from 2000 to 2017. During this period *Sp* and *Hi* represented 27.51% of cases and other pathogens accounted for 5.35% of all meningitis cases only. 30 CSF samples which had Gram strains compatible with Nm but with no growth on culture, and in which PCR was not done represented 0.26% for the period 2000 to 2017. Overall, meningitis cases were highly dominated by Nm with different strains over the period, with NmC and NmW dominating in terms of confirmed cases and progressively replacing NmA in both time and space.

#### Temporal distribution of bacterial meningitis agents

**Fig. 3:**
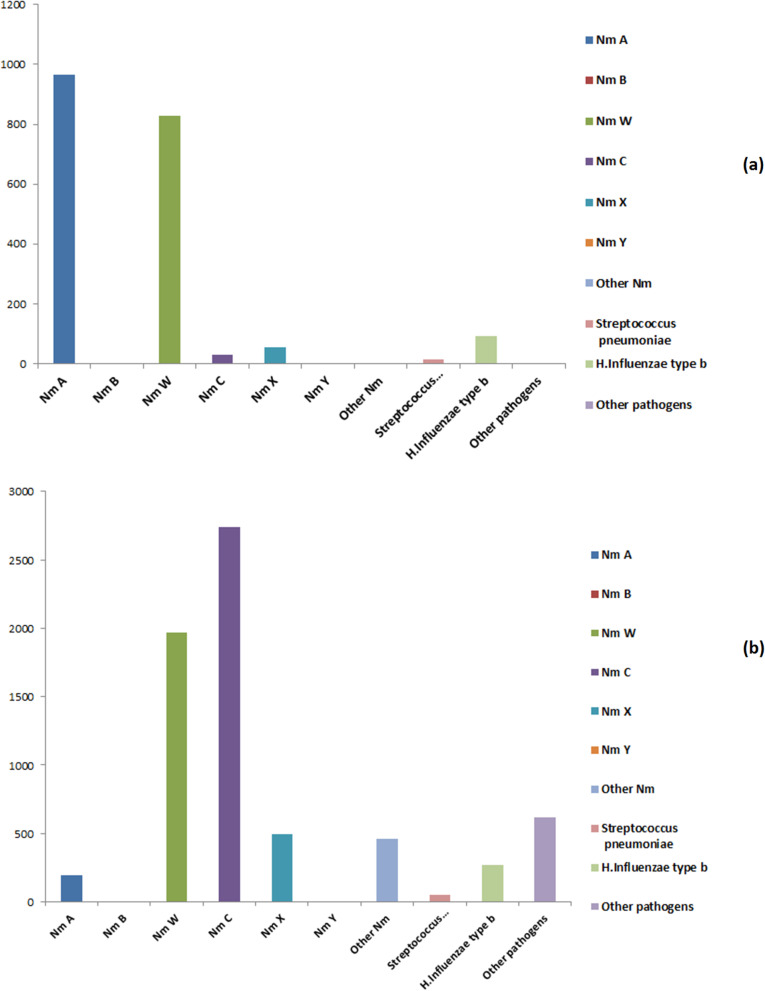
Time-series of bacterial meningitis pathogens identified in Africa from 2000 to 2010 (**a**) and from 2011 to 2017 after MenAfrivac (**b**)

Since 2010, corresponding to the progressive introduction of MenAfrivac vaccine in Africa, there has been a downward trend in notifications of NmA. NmW and C and *Sp* increased from 2010. The NmC strain showed a peak of case notifications between 2015 and 2017, and thus appears to have significantly increased in occurrence in Africa following the introduction of MenAfriVac. The *Sp* pathogen, for its part, also seems to be increasing in Africa over this period and experienced a peak of case notifications in 2016 and in 2017 (Fig. 3 and Table 1).

#### Spatial distribution of bacterial meningitis agents

**Fig. 4:**
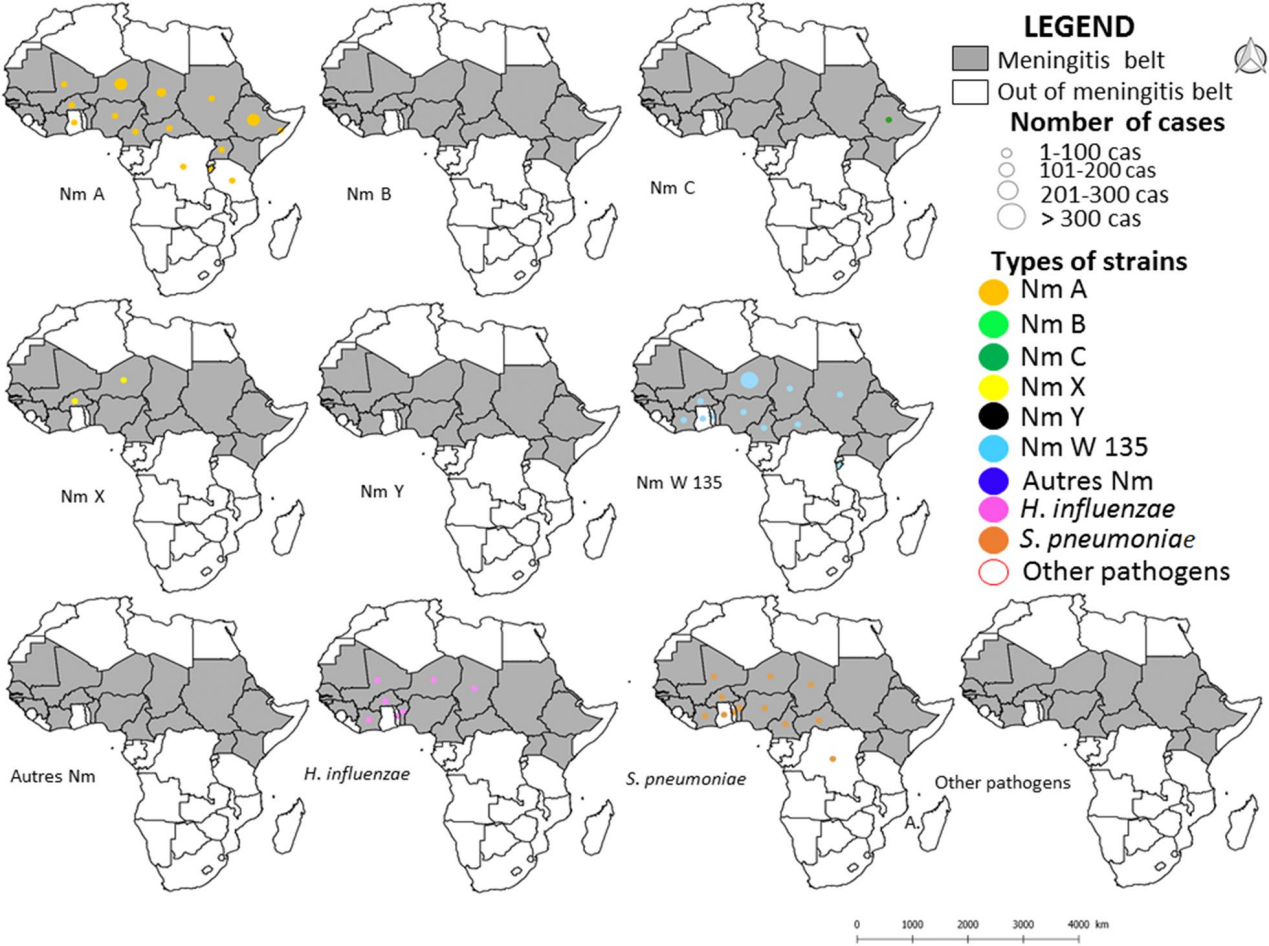
Spatial distribution of different pathogen species and strains causing meningitis in Africa by country from 2000 to 2010

**Fig. 5:**
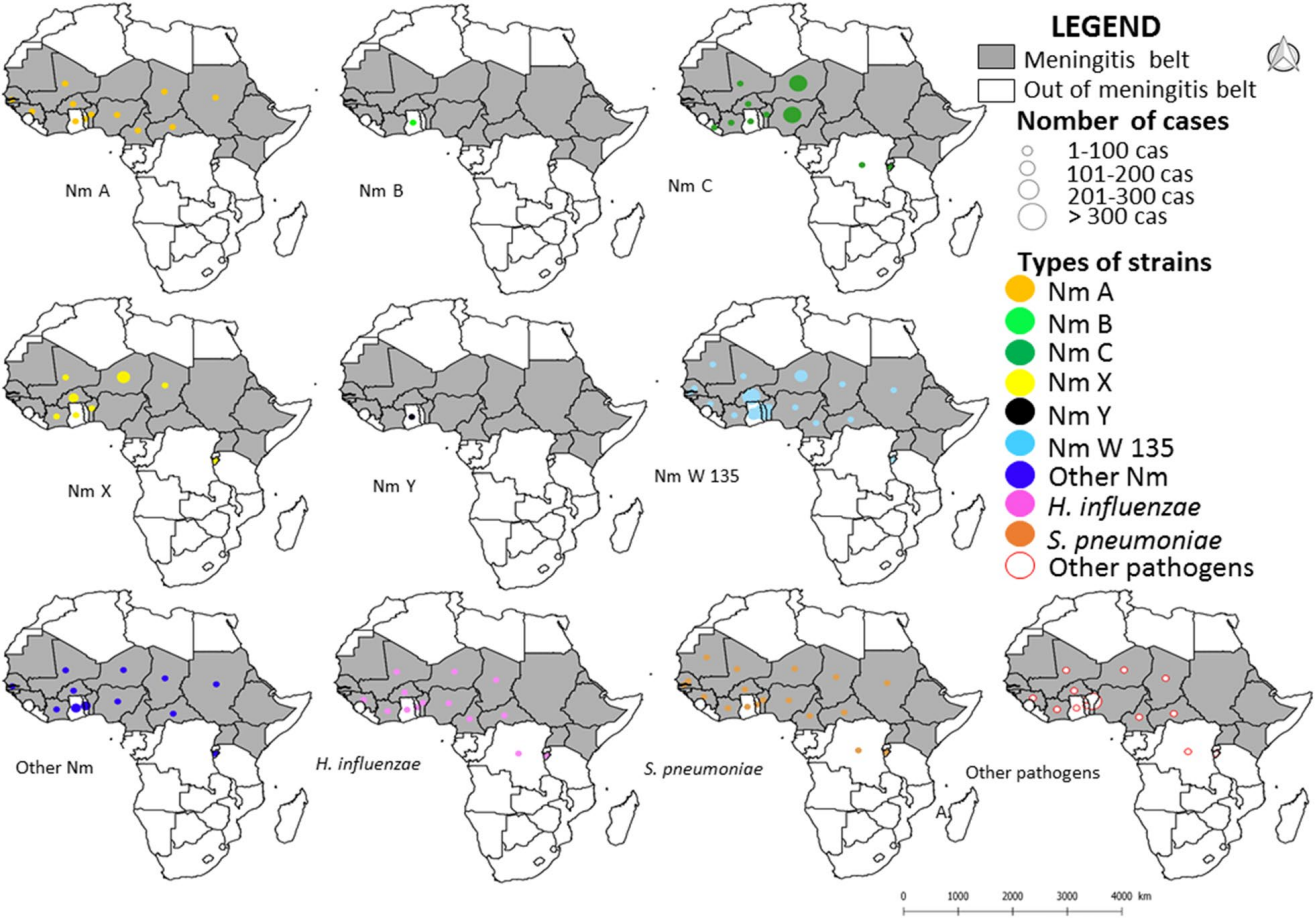
Spatial distribution of different pathogen species and strains causing meningitis in Africa by country from 2011 to 2017

Between 2000 and 2017, it is evidenced that Western African countries, particularly Niger, Nigeria and Burkina Faso (three countries of the meningitis belt) and a country in Eastern Africa, i.e., Burundi, and one in central Africa, i.e., DRC, both located outside the belt, experienced a greater diversity of circulating bacterial strains causing meningitis: six distinct bacterial species or groups for Niger, Burkina Faso and Burundi (NmA, NmC, NmW, NmX, Sp which has also serotypes and Hi), and 5 for Nigeria and DRC (NmA, NmC, NmW, Sp and Hi). Specifically, these five African countries appear today as an important cradle for the circulation of co-existing strains of bacteria causing meningitis (Figs. 4 and 5). The differences observed in the spatial distribution of pathogen strains as listed on Figs. 4 and 5 and the spatial distribution of meningitis cases (see Fig. 2) correspond to a significant absence of clinical identification for a very large number of situations and countries.

## Discussion

Meningitis, in its epidemic phases, has strongly affected Africa since the XXth century [3, 15, 40]. Meningitis outbreaks occurred well before modern times and African communities must have suffered from severe epidemics of meningitis in the past. However, the first censuses of meningitis cases date from this time, i.e., 1909 on the West coast of Africa [3]. Meningitis epidemics usually affect countries in Sub-Saharan and Sudanian Africa grouped under the so-called "African meningitis belt" (or Lapeyssonnie belt, named after the French military medical doctor who described it in 1960) [11, 40, 41].

Known as a characteristic infectious disease of the Sahelo-Sudanian region in Africa, punctuated by the breath of Harmattan winds [3, 42–44], the persistence of long dry seasons and the effect of desert aerosols made of dust and sand particles are important environmental and ecological determinants that have been put forward to explain epidemic development phases of meningitis epidemics within the belt [17, 19, 45–49].

Since 1905, there has been a multiplication of meningitis epidemics in Africa, but also in countries outside the traditional so-called meningitis belt, often located in remoted regions of Africa (East, Central and South), and which were not referenced as centers of meningitis notifications before. This continent-wide spread of meningitis confirms its status as an emerging or re-emerging infectious disease (EID) in Africa [15] although it is not fully classified as such, and a neglected tropical disease (NTD) due to the less health consideration currently made for infected countries located outside-the-belt (the present work).

The epidemics of bacterial meningitis reported outside the belt inevitably have not been well studied, and undoubtedly this study may suffer from serious sampling biases. It is important to improve the methods of collecting and reporting data relating to meningitis and thus improve their quality for all countries. Definitely, this will allow us to improve the quality of future studies, but unfortunately this is the state of the current situation for both this EID and NTD. However, this is where we are with a lack of international recognition today of the existence of important meningitis epidemics outside its well-known historical spatial distribution.

Definitely this study may suffer from some limitations on the completeness of data especially on disease etiology used in this review which is known to suffer from underreporting and potential sampling biases [50]. The isolation data are problematic as there is little consistency in the taking of CFS samples or culturing throughout Africa, due to resource and capacity constraints. These limitations could be improved in subsequent studies, and should constitute a medical and research priority for the near future. However, this first synthesis on bacterial meningitis on the African continent for a long period not only makes it possible to contribute to the knowledge of meningitis burden and spatio-temporal patterns in Africa but especially to highlight the often-emerging and still neglected hypothesis of a spatial and progressive trend towards the gradual "out-of-the-belt" widening of the actual distribution area of meningitis epidemics in Africa [4, 42]. Through our overview study, we have been able to highlight a steady increase of meningitis epidemics on the African continent during the XXth century, with a very large majority of African countries being affected and being actually out of the radar of international public health programs.

Year-1996 represents the period of time in which Africa experienced the highest number of cases of bacterial meningitis, with a threshold reached of 180,000 cases [51]. This makes it not only an emerging or re-emerging disease but also one of the most worrying infections for the health of African citizens today.

On the basis of the cases reported by the different countries located outside the African meningitis belt, our work highlights that three countries, Ghana, the Democratic Republic of Congo and Egypt, constitute during the XXth century the most affected countries by meningitis epidemics in this new extension outside-the-belt zone. To a lesser extent, Tanzania and Burundi may also constitute two important countries to be systematically surveyed and diagnosed for these types of bacterial infections in human.

Although the geographic spread of meningitis epidemics outside the meningitis belt is convincing, the regions located inside the belt still stay the most affected. Indeed, more than three quarters of the cases of bacterial meningitis reported in Africa from 1928 to 2018, or 84.8%, still occur in the meningitis belt [4, 6, 52]. This constitutes a rationale for still accepting the existence of the meningitis belt between the parallels 10° N and 15° N. Nevertheless, accepting that meningitis is today spreading all over the African continent, and particularly in Central and Eastern Africa should be accepted as a regional and international health priority.

Four hypotheses could account for the geographic extension of meningitis epidemics outside the meningeal belt. The first hypothesis concerns the links that exist between the current climatic disturbances and their consequences on African ecosystems, and consequently on the most incriminated bacterium *N*. *meningitidis* and its adaptation to new environmental contexts favorable for its development and spread [53–57]. The second is an anthropogenic hypothesis related to the continuous and accelerated deforestation and fragmentation of tropical rainforest areas in Central Africa for half a century, notably in the African central basin, and which is adjacent to forested regions of Southern Africa. These important large-scale environmental perturbations would cause an immense corridor for the circulation and spread of germs responsible for infectious diseases including meningitis [58–60]. This has been particularly well documented recently with the example of the tropical infectious disease Buruli ulcer caused by *Mycobacterium ulcerans*, which is benefitting from man-made deforested landscapes to proliferate and cause new infections in human communities [61]. A third hypothesis concerns the lack of assistance and the strengthening of sanitary capacities to fight against meningitis, despite the fantastic progress that has been made in the most recent years [62]. The fourth hypothesis could be constituted by migrations from north to south of human populations.

A total of 2,628,283 cases and 151,808 deaths caused by meningitis were reported by 53 African countries from 1928 to 2018, which places meningitis as the leading bacterial cause of morbidity and mortality in children by its importance and severity on the African continent [63]. The average lethality rate of 5.7% over the period covered by our present work (1928–2018) indicates that this infectious disease is still a scourge for many African populations. We estimate that this average rate is very heterogeneous from one region of Africa to another and it should mirror what we observe at the grain of one central African country, i.e., DRC, that has been studied recently [64], and is very probably underestimated, particularly in view of the weakness in the completeness of the current recorded data, itself dependent on precariousness of surveillance systems in some areas, and the inherent difficulties to proceed to clinical and laboratory confirmations. Diagnostic capacity is different between countries of the belt, and they also receive a lot of support from WHO and others to strengthen meningitis diagnosis. Contrastly, countries outside of the belt receive very little or no funding support that can also constitute a bias in the data reported [26].

The diversity of bacterial strains and species responsible for meningitis epidemics in Africa is another important explanatory factor for the uncertainty surrounding lethality. Indeed, in a study of the meningitis epidemics of the meningeal belt published in 2002, Chippaux and his collaborators showed that lethality due to meningitis varies according to bacterial strains and species [42]. According to WHO estimates, the lethality of meningitis caused by NmA is 5 to 10% [27] but the estimated lethality for other strains is still absolutely unclear. The high frequency of NmA may also explain why it is the most studied strain to date, and the one that remains the most representative of the symptoms responsible for meningitis [65].

Since the massive introduction of meningococcal serogroup A conjugate vaccine (MenAfriVac) in 2010, a transitional change in the epidemiology of meningitis epidemics in Africa has occurred as illustrated in the present work, with a dramatic decrease in the number of outbreaks caused by NmA [66]. This decrease is particularly marked in some countries, for example in the case of Niger, Western Africa where NmA serogroup has disappeared while serogroups NmC, NmX, and NmW are now responsible for recurrent epidemics in this country. Our study indicates that since 2010 a downward trend in the reporting of NmA has begun. With the largest case peak in 2010, strain NmC was the most common from 2015 to 2017 in Africa. The NmW strain coexisted with the latter from 2010 to 2012. The bacterial species *S. pneumoniae* was also prevalent in 2016, and in 2017 the strain NmX was responsible for a large case peak. The increasing trend of other strains of *N. meningitidis* (other than NmA) is currently worrying [7], in particular because it seems that the elimination of NmA by vaccination may have led to its almost immediate replacement in human populations by other bacterial strains, as in an ecological process of occupation of ecological niches rendered empty or the existence of ecological interference and competition across bacterial strains. Increased surveillance of multiple serogroups throughout the African region is absolutely necessary, as well as consideration of vaccination with combination vaccines rather than just using a single strain as is currently the case against NmA [67–69]. Definitely this constitutes a priority challenge in the future to control these meningitis multi-strain infections if we want to decrease the burden of bacterial diseases due to the three main bacteria (*Neisseria meningitidis, Haemophilus influenzae* and *Streptococcus pneumoniae*) in Africa in the near future.

## Conclusion

The present study has shown that the burden of meningitis is still high in Africa today, and progress in control is significantly lower than for other vaccine-preventable diseases [62, 70, 71]. Special attention needs to be paid to the development of vaccines with wider coverage against the different pathogens responsible for meningitis epidemics. Efforts should be made to make these vaccines available to the health services in the most affected countries, to increase people's acceptance of these vaccines, and finally to improve access to diagnostics and treatment in case of outbreaks [62]. Meningitis remains a major public health problem in Africa. Formerly concentrated in the region of the meningitis belt with desert or sub-desert climates and other similar environmental conditions, this disease extends today outside these historical limits to reach forested regions located at the heart of Africa. With environmental changes undergoing today, an epidemiological transition of meningitis appears to be taking place, requiring a better consideration of the etiological nature of the responsible agents, coupled with cross-cutting and integrative studies on the ecology and the evolution of the transmission of these African meningitis outbreaks of different etiological natures. The challenges are many, certainly difficult, but by associating different disciplines and adopting an approach of ecology and evolution of health, the so-called *One Health* and *Ecohealth* approaches, we will better understand the different determinants responsible for the maintenance and the epidemic propagation of not only meningitis but also other infectious diseases like Ebola virus disease, measles and pertussis among others in Africa. Inside-the-belt meningitis is still definitely true, but the international health community should be aware that bacterial meningitis has progressively reached regions outside-the-belt, and that current time is ringing the bell for a better transcontinental public health surveillance and action. Strengthening the health surveillance system all over the 54 African countries, and not only countries belonging to the meningitis belt, we can then expect to improve the regional capacities to fight against this severe disease.

## Supplementary Information

**Additional file 1.** Database_of_meningitis_confirmed_cases_2000_2017.

**Additional file 2.** Number_of_cases_meningitis_8_years_5.

## Data Availability

All data generated or analysed during this study are included in this published article [and its supplementary information files]. All the data accessible by the links below are publicly/ freely accessible: https://apps.who.int/iris/handle/10665/229717?search-result=true&query=Meningitis+data+since+1928+in+Africa&scope=%2F&rpp=10&sort_by=score&order=desc. (Weekly Epidemiological Record 1930) (accessed 9.5.19). https://apps.who.int/iris/handle/10665/72037 (accessed 9.5.19). https://www.who.int/csr/don/archive/disease/meningococcal_disease/en/ (accessed 9.5.19). http://www.who.int/gho/epidemic_diseases/meningitis/epidemic_districts_text/en/ (accessed 9.5.19). http://www.safetravel.ch/safetravel2/servlet/ch.ofac.wv.wv203j.pages.Wv203ActualitesCtrl?action=afficheDetail&refActu=000437 (accessed 9.5.19). https://www.who.int/emergencies/diseases/meningitis/epidemiological/en/ 1 (accessed 10.5.19). https://www.who.int/csr/resources/publications/meningitis/whoemcbac983.pdf?ua=1 (accessed 9.5.19). https://apps.who.int/iris/bitstream/handle/10665/72037/bulletin_supp%20_Vol28.pdf?sequence=1&isAllowed=y (accessed 9.5.19). http://promedmail.chip/mailman/listinfo/promed and http://www.flutrackers.com/forum) (accessed 9.5.19).

## Abbreviations

ACF: Autocorrelation function
AR: Autoregressive
ARMA: Autoregressive moving average
CSF: Cerebrospinal fluid
*df*: *degree of freedom*
DRC: Democratic Republic of Congo
EPI: Expanded Programme on Immunization
Hia: *Haemophilus influenzae type a*
Hib: *Haemophilus influenzae type b*
HZ: Health zone
IDSR: Integrated Diseases Surveillance System
INRB: Institut National de Recherche Biomédicale
MA: Moving average
Nm: *Neisseria meningitidis*
*p*: P-value
PCR: Chain Reaction
PEV: Programme Elargi de vaccination
S.D: Standard deviation
Sp: *Streptococcus pneumonia*e
W: Week
WHO: World Health Organization
